# Efficacy and safety of a 4-year combination therapy of growth hormone and gonadotropin-releasing hormone analogue in pubertal girls with short predicted adult height

**DOI:** 10.3389/fendo.2023.1113750

**Published:** 2023-03-17

**Authors:** Hilde Dotremont, Annick France, Claudine Heinrichs, Sylvie Tenoutasse, Cécile Brachet, Martine Cools, Kathleen De Waele, Guy Massa, Marie-Christine Lebrethon, Inge Gies, Jesse Van Besien, Christine Derycke, Mathieu Ziraldo, Jean De Schepper, Véronique Beauloye, Stijn Verhulst, Raoul Rooman, Marieke den Brinker

**Affiliations:** ^1^ Department of Pediatrics, University Hospital Antwerp, Edegem, Belgium; ^2^ Laboratory of Experimental Medicine and Pediatrics, University of Antwerp, Edegem, Belgium; ^3^ Hôpital Universitaire des Enfants Reine Fabiola, Université Libre de Bruxelles, Brussels, Belgium; ^4^ Department of Pediatric Endocrinology, Department of Internal Medicine and Pediatrics, Ghent University, Ghent University Hospital, Ghent, Belgium; ^5^ Department of Pediatrics, Jessa Hospital, Hasselt, Belgium; ^6^ Department of Pediatrics, University Hospital of Liège, Liège, Belgium; ^7^ Department of Pediatric Endocrinology, University Hospital Brussels, Brussels, Belgium; ^8^ Belgian Society for Pediatric Endocrinology and Diabetes (BESPEED), Brussels, Belgium; ^9^ Unité d ‘Endocrinologie Pédiatrique Cliniques Universitaires Saint Luc, Université Catholique de Louvain, Brussels, Belgium; ^10^ PendoCon, Putte, Belgium

**Keywords:** short stature children, girls, growth hormone treatment (GH), gonadotropin-releasing hormone agonist (GnRHa), adult height, puberty

## Abstract

**Objectives:**

To improve adult height in pubertal girls with a poor height prediction, treatment with growth hormone (GH) can be used in combination with a gonadotropin releasing hormone agonist (GnRHa), to delay closure of the growth plates. However, there are few studies to support this practice, and they show conflicting results. The objective of this trial is to assess the safety and efficacy of this combination treatment in early pubertal girls with a short predicted height, in comparison with matched controls.

**Design, patients, and methods:**

We designed an open-label, multicenter, interventional case-control study. Early pubertal girls with predicted adult height (PAH) below -2.5 SDS, were recruited in tertiary care centers in Belgium. They were treated for four years with GH and GnRHa. The girls were followed until adult height (AH) was reached. AH *vs* PAH, AH *vs* Height at start, and AH *vs* Target Height (TH) were evaluated, as well as safety parameters. Control data were assembled from historical patient files or from patients who preferred not to participate in the study.

**Results:**

Sixteen girls with mean age ( ± SD) at start of 11.0 years (± 1.3) completed the study protocol and follow-up. Their mean height ( ± SD) increased from 131.3 ± 4.1 cm (-2.3 ± 0.7 SDS) at start of treatment to 159.8 ± 4.7 cm (-1.1 ± 0.7 SDS) at AH. In matched controls, height increased from 132.3 ± 4.2 cm (-2.4 ± 0.5 SDS) to 153.2 ± 3.4 cm (-2.1 ± 0.6 SDS) (p<0.001). AH surpassed initial PAH by 12.0 ± 2.6 cm in treated girls; and by 4.2 ± 3.6 cm in the controls (p<0.001). Most treated girls reached normal adult height (>-2SD) (87.5%) and 68.7% reached or superseded the target height (TH), which was the case in only a minority of the controls (37.5% and 6.2%, respectively) (p= 0.003 and 0.001). A serious adverse event possibly related to the treatment, was a fracture of the metatarsals.

**Conclusion:**

A four-year GH/GnRHa treatment in early pubertal girls with a poor PAH seems safe and results in a clinically relevant and statistically significant increase in AH compared with matched historical controls.

**Clinical trial registration:**

ClinicalTrials.gov, identifier NCT00840944.

## 1 Introduction

Many short children and their families ask for an adult height (AH) prediction at the beginning of puberty. If this prognosis falls below their expectations, they often inquire about methods to increase AH by maximizing their pubertal height gain. Unfortunately, the remaining height gain at the beginning of puberty represents only 15-20% of total height. Closure of growth plates limits the intervention time (1, 2). This process is driven by gonadal steroids, mainly estrogens (3, 4). Gonadotropin-releasing hormone analogues (GnRHa) efficiently suppress the progression of puberty and delay the closure of growth plates. In girls with precocious puberty, GnRHa treatment results in a gain in AH of 3 to 10 cm (5). In children with normal pubertal timing but with poor AH prognosis the results of GnRHa treatment are rather disappointing, with a gain in AH ranging from 1 to 4.2 cm (6–8). These modest results can be explained by the fact that GnRHa not only slow down skeletal maturation but also reduce growth hormone secretion and growth velocity (9).

To prevent this slowdown of growth velocity during GnRHa therapy, a combined treatment of GnRHa with growth hormone (GH) has been explored in different clinical settings. In girls with precocious puberty, AH minus predicted adult height (PAH) at start was higher in girls treated with combined GH/GnRHa therapy (4.7 to 11.4 cm) compared with girls treated with GH alone (10–13). In children with GH deficiency (GHD) who entered puberty early, Tauber et al. reported no difference in AH minus PAH between the combined GH/GnRHa treatment and the GH-only treatment, although the duration and dosage of GH therapy was variable (14). Mericq et al. however, found a significant increase in near-adult height (NAH) after three years of combined GH/GnRHa treatment compared with GH alone in GHD (15). The first reports on this combined GH/GnRHa treatment in children with idiopathic short stature (ISS) did not show any improvement of AH compared with controls (16, 17). The National Cooperative Growth Study (USA) found that NAH exceeded initial PAH with 3 ± 6.1 cm in a pooled group of patients with GHD or ISS treated with combined GH/GnRHa treatment (18).

More recent studies with higher GH doses and longer treatment durations in children born small-for-gestational age (SGA) or with ISS reported a difference in AH *vs* PAH of 4.9 to 10.8 cm (19–26). Bennabad et al. found no difference in NAH-SDS between the combined GH/GnRHa treatment and the GH alone treatment in ISS children after 2.4 years, but the study was prematurely discontinued (27). With this longer duration of treatment however, by postponing puberty development for such a long time, psychosocial functioning can be impaired (28). Significant better height gains of combined GH/GnRHa treatment have also been reported in short adopted girls (29, 30), and patients with SHOX deficiency (31) (**Supplementary Table 1**).

The level of evidence of many of these studies, however, was limited due to either small numbers of patients, the lack of a control group or a short follow-up time (32). Furthermore, most studies did not consider the limited accuracy of height prediction in this group of patients. Therefore, we conducted a prospective, case-controlled clinical trial of four-year combination treatment with GH and GnRHa in a cohort of girls with ISS at the beginning of puberty and with follow-up to AH.

## 2 Subjects and methods

### 2.1 Trial design

This open-label, multi-center study ran in 6 Belgian tertiary pediatric endocrinology units. The protocol was approved by the central ethics committee of University Hospital Antwerp and the local ethics committees of the participating centers. The clinical trial was registered in the EU Clinical Trials Register under (EudraCT 2007-003247-70) and at ClinicalTrials.gov. (NCT00840944). Informed consent/assent was obtained from both parents and participating children. The participants were treated for 48 months and then followed until AH was attained.

At the start of the trial, in the original protocol set-up, boys and girls were included. During the inclusion period however, only 6 boys were recruited. Apparently, the long period of puberty postponement seemed to be a psychologic burden too high for them to start. Of these 6 boys, 4 completed the treatment phase, and only 1 completed the follow-up period. Therefore, we decided to exclude boys from further analysis.

### 2.2 Inclusion and exclusion criteria

We included girls with early pubertal stage (breast stage B2-B3), a bone age between 10 and 12 years, a height prediction < 151.0 cm (-2.5 SDS on Flemish growth charts) (33), normal body proportions (sitting height/height between -2 SDS and + 2 SDS) (34) and IGF-I within the reference range as provided by center-specific immune assays. We excluded girls who were adopted, or had syndromic short stature, chronic disease or chronic use of medication that is known to interfere with growth. Girls with Tanner puberty stage B3 or a small birth weight and/or birth length were not excluded, so that the study would reflect the real world population in our clinics.

### 2.3 Historical controls

For each patient that completed the full study and follow-up phase, a control was sought from historic patient files or among the children who met the inclusion criteria but preferred not to use growth-promoting treatment. Controls were matched for 3 predefined characteristics considered to be of influence on the outcome: (1) bone age at the moment of growth prediction (≤ 11 years or >11 years); (2) degree of short stature, calculated as the PAH (≤147.0 cm or > 147.0 cm) and (3) PAH SDS minus Target Height (TH) SDS (< 1.0 or ≥ 1.0). The controls were not systematically followed in the study and were invited to the clinic for determination of their AH.

### 2.4 Interventions

The participating girls were treated with a 4-year combination therapy of GH (Zomacton^®^, Ferring) and the GnRHa triptorelin acetate (Gonapeptyl SR^®^, Ferring). GH was given as a daily SC transjection of 0.05 mg/kg body weight. The GH dose was adjusted to bodyweight at each visit unless IGF1-levels exceeded center-specific references. Triptorelin was administered IM or SC at a dose of 3.75 mg every 4 weeks.

### 2.5 Patient evaluations

Patients were evaluated at start, 3 and 6 months, and every 6 months thereafter until the end of treatment, then yearly until AH was reached.

Height was measured using a Harpenden stadiometer and sitting height using a Harpenden Sitting Height Table. Height SDS were calculated using Flemish growth reference data (33). Bone age was determined yearly by the local investigator using the Greulich and Pyle method (35) and PAH was calculated using the “average” Bayley and Pinneau tables (36). AH was defined as the standing height attained at a bone age of 16.0 years or more. AH SDS was calculated using the mean and SDS for 21-year-old females in the Flemish population study. Puberty was staged according to the method of Tanner (37). TH was calculated according to Tanner as [paternal height (cm) – 13 cm + maternal height (cm)]:2 (38).

The following laboratory tests for safety and compliance were performed at the local hospital at baseline and then yearly: full blood count, liver- and kidney function tests, Free T4, fasting blood glucose and insulin, HbA1c, LH, FSH, estradiol and IGF-I.

### 2.6 Data handling and statistics

The investigators arbitrarily decided that a difference of at least 6 cm was needed to make this intervention clinically and ethically meaningful. With an expected difference in adult height of 6 cm, a standard deviation of 6 cm in a T test for independent variables, with a type I error of 0.05 and a power of 80%, 16 subjects must be present in each group (Dupont and Plummer 1990). Because of the long trial duration, a 30% drop-out was assumed. Some patients missed a visit during the follow-up phase. In those cases, height data were linearly interpolated from the previous and following visit. All statistical analyses were performed using SPSS version 27.0 (SPSS, Inc., Chicago, IL, United States). Measurement data were expressed as mean ± SD (standard deviation) if normally distributed, and with median ± IQR if not normally distributed. Normality was tested by the Kolmogorov-Smirnov test. Variables that showed a normal distribution were analyzed by an independent samples t-test; not normally distributed variables by the Mann-Whitney U test. P-values of ≤ 0.05 were considered statistically significant. Chi-square test was used to compare observed to expected results for TH and AH. To evaluate the influence of baseline characteristics on the treatment effect parameters – AH, AH-PAH and AH- H_0_ – we conducted Spearman’s correlation with the treatment effect parameters as primary dependent variables. Two-tailed p values < 0.05 were considered statistically significant.

*Post hoc*, a sensitivity analysis was performed to assess the effect of a more advanced puberty stage (B3) and a small size at birth on the results. For this analysis, the patients with a B3 pubertal stage were removed from the active group and the patients with SGA in the control group were replaced by non-SGA controls.

## 3 Results

### 3.1 Population

**Figure 1:**
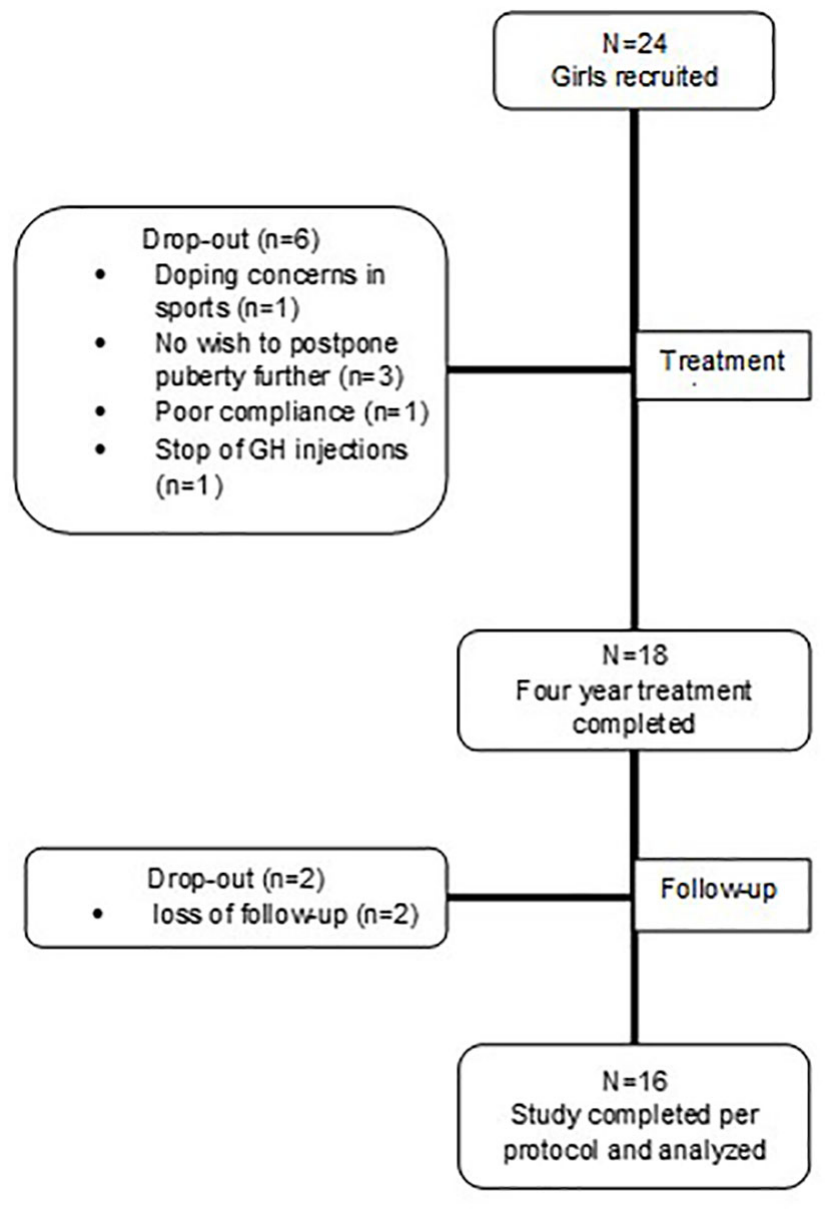
Flowchart presenting patient disposition.

Twenty-four girls were recruited and received at least one dose of study medication (intention-to-treat (ITT) group). Eighteen girls completed the 4-year treatment phase, and 16 girls completed the follow-up to AH (per protocol (PP) group). Reasons for drop out were doping concerns in sports (n=1), no wish to postpone puberty any longer after 2.5 or 3 years (n=3), poor compliance (n=1) and premature stop of GH injections (n=1; after 42 months). Two girls were lost to follow-up 12 to 36 months after the treatment phase (**Figure 1**). In summary, one third of the girls who initiated treatment were excluded from the study analysis because of preliminary drop-out.

To evaluate the potential bias of poor responders being the dropouts, we determined besides height gain at drop-out also mean height gain at the same time-point in the PP group (**Table 1**). This shows that for the drop-out patients, the height gained at the last visit, was comparable to the height gain in the PP group at that same time point, thus not suggestive for an exclusion bias. Of those with decreased height gain, patient V was very short, and had a very short mother (136 cm). She did not respond well to the treatment and stopped because of a lack of treatment effect. We suspect an underlying genetic cause of her growth faltering.

**Table 1:** Height gain attained at the last available visit in patients who dropped out compared to height gained in the PP group at the same visit.

	Reason for exclusion	Last available visit (months)	Height gain (cm)	Height gain in the PP group (mean ± SD)
**I**	compliance due to difficult family situation	60	24.8	26.3 ± 4.0
**II**	Compliance due to difficult social situation	60	23.3	26.3 ± 4.0
**III**	patient wished to stop	24	15.3	14.7 ± 2.3
**IV**	patient wished to stop	36	18.2	19.8 ± 3.3
**V**	patient wished to stop	42	14.5	22.0 ± 3.6
**VI**	patient wished to stop	36	27.9	19.8 ± 3.3
**VII**	patient wished to stop	24	14.2	14.7 ± 2.3
**VIII**	patient wished to stop	30	13.2	17.2 ± 2.8

### 3.2 Baseline characteristics

Clinical and baseline characteristics of study patients (PP group) and matched controls are shown in **Table 2**. These parameters did not significantly differ between the two groups, however, matched controls tended to have a little higher height prediction than treated girls (+1.3 cm) (P=0.075). Most girls were early pubertal (breast stage 2), only one girl in the treatment group and one girl in the control group had a breast stage 3. Three girls in the control group were born SGA. Four of the treated girls and 3 of the controls had a TH SDS ≤ -2. In all treated girls and controls, at the time of screening blood tests were normal, including IGF-I within center-specific reference ranges. Baseline characteristics for the group that was used in the sensitivity analysis are shown in **Supplementary Table 2**.

**Table 2:** Clinical characteristics and outcome parameters of treated girls in comparison to matched controls (mean ± SD).

Clinical characteristics	GnRHa +GH treated girls (n=16)	Matched controls (n=16)	P
**Age at start (y)**	11.0 ± 1.3	11.5 ± 0.9	0.277
**Birth Length (cm)**	46.9 ± 1.6	46.0 ± 4.1	0.808
**Birth Weight (g)**	2787 ± 294	2610 ± 638	0.968
**Gestational age (w)**	38.6 ± 1.6	38.8 ± 2.6	0.773
**TH (cm)** **(SDS)**	157.7 ± 5.3 -1.4 ± 0.8	159.3 ± 4.5 -1.2 ± 0.7	0.366 0.311
**H_0_ (cm)** **(SDS)**	131.3 ± 4.1 -2.3 ± 0.7	132.3 ± 4.2 -2.4± 0.5	0.409 0.307
**H_0_- TH (cm)** **(SDS)**	-26.4 ± 4.3 -0.7 ± 1.1	-26.8 ± 6.2 -1.1 ± 1.1	0.832 0.186
**BA at start (y)**	10.5 ± 0.6	10.4 ± 0.5	0.969
**PAH (cm)** **(SDS)**	147.8 ± 2.0 -3.2 ± 0.5	149.1 ± 2.2 -2.8 ± 0.4	0.075 0.053
**AH (cm)** **(SDS)**	159.8 ± 4.6 -1.2 ± 0.7	153.2 ± 3.4 -2.1 ± 0.6	<0.001 <0.001
**AH-PAH (cm)** **(SDS)**	12.0 ± 2.6 2.0 ± 0.7	4.2 ± 3.6 0.7 ± 0.5	<0.001 <0.001
**AH-TH (cm)** **(SDS)**	1.7 ± 5.4 0.4 ± 0.9	-6.1 ± 4.5 -0.9 ± 0.8	<0.001 <0.001
**AH– H_0_ (cm)** **(SDS)**	28.4 ± 4.2 1.1 ± 1.0	21.3 ± 4.3 0.4 ± 0.7	<0.001 0.033

TH, target height; Ho, height at the start of treatment; BA, Bone age; AH, adult height; PAH, predicted adult height.

### 3.3 Height evolution

**Figure 2:**
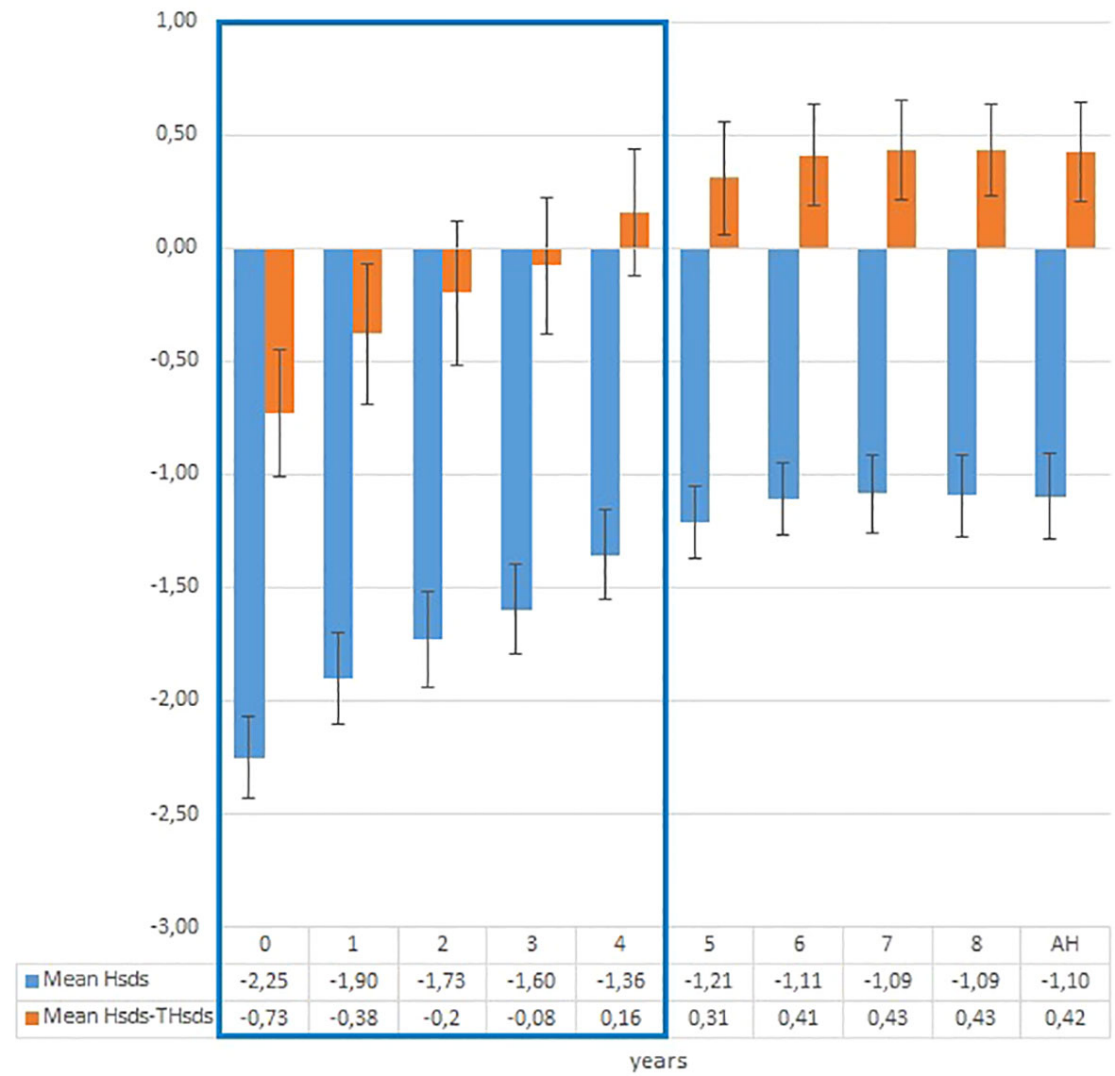
Evolution of mean height SDS and mean height SDS-target height SDS in treated patients during treatment and follow-up phase.

In the PP group, mean height (± SD) increased from 131.3 (± 4.1 cm) to 155.3 (± 4.7 cm) at the end of treatment and to 159.8 (± 4.7 cm) at AH. Mean height SDS increased from -2.3 (± 0.7) at the start of treatment to -1.4 (± 0.8) at the end of the treatment and to -1.1 (± 0.7) at AH (**Figure 2**). In the control group, mean height (± SD) increased from 132.3 (± 4.2 cm) to 153.2 (± 3.4 cm) at AH. Mean height SDS in controls increased from -2.4 (± 0.5) to -2.1 (± 0.6) at AH. AH was significantly higher in the PP group compared with the controls (p<0.001).

Mean absolute height gain (± SD) – expressed as AH-H_0_ – was 28.4 (± 4.2 cm) in the treated patients and 21.3 ( ± 4.3 cm) in the controls (p<0.001).

The proportion of girls who attained normal AH (AH SDS >-2) was 87.5% in PP group and 37.5% in the control group (chi-square p=0.003). Eleven out of sixteen (68.7%) treated girls and one out of sixteen (6.2%) controls reached AH equal or higher than TH (chi-square p= 0.001).

**Figure 3:**
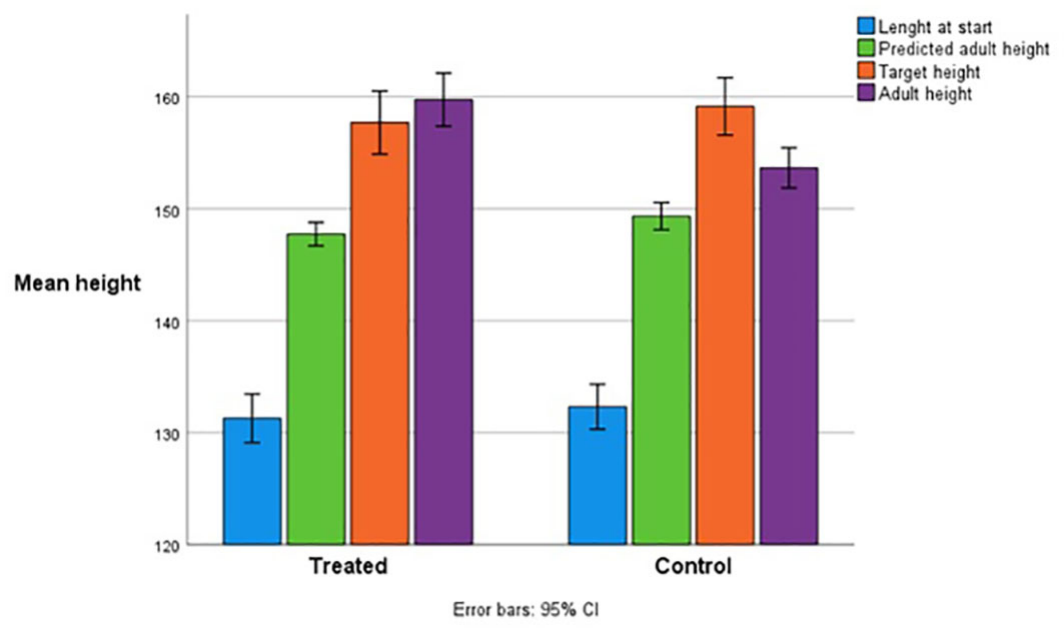
Adult height compared with predicted adult height and target height in the treated and control patient group.

In the PP group, height surpassed initial PAH by 7.6 ± 3.9 cm at the end of treatment and by 12.0 ± 2.6 cm at AH (**Figure 3**). In controls, AH surpassed the initial PAH by 4.2 ± 3.6 cm (p<0.001). In the PP group, PAH at the end of treatment (162.4 ± 5.5 cm) was 14.8 ± 4.2 cm above PAH at the start of treatment (147.7 ± 1.9 cm). However, actual AH was 2.6 cm shorter (159.8 ± 4.6 cm) than PAH at the end of treatment. The PAH after 3 and 4 years of treatment was similar: 160.0 +/- 5.5 cm after 3 years and 160.9 +/- 5.5 cm after 4 years.

In the PP group, AH correlated significantly with PAH at start of treatment (p= 0.046; r=0.503); and height gain SDS correlated significantly with height (p=0.016; r=-0.589), age (p=0.001; r=0.755) and bone age delay (p=0.001; r=0.797) at start of treatment. However, height deficit in relation to TH at start of treatment (H SDS-TH SDS) did not correlate with the outcome parameters AH, AH-PAH and AH-H_0._ Finally, in all girls, treated and controls, the outcome parameters – AH, AH-PAH and AH-H_0_ – did not significantly correlate with the baseline parameters such as birth weight, birth length, gestational age, maternal height, paternal height, TH, and bone age at start of therapy.

The results of the *post hoc* sensitivity analysis are shown in **Supplementary Table 2**. The elimination of the puberty stage B3 and the SGA girls did not substantially change the results.

### 3.4 Bone age progression

**Figure 4:**
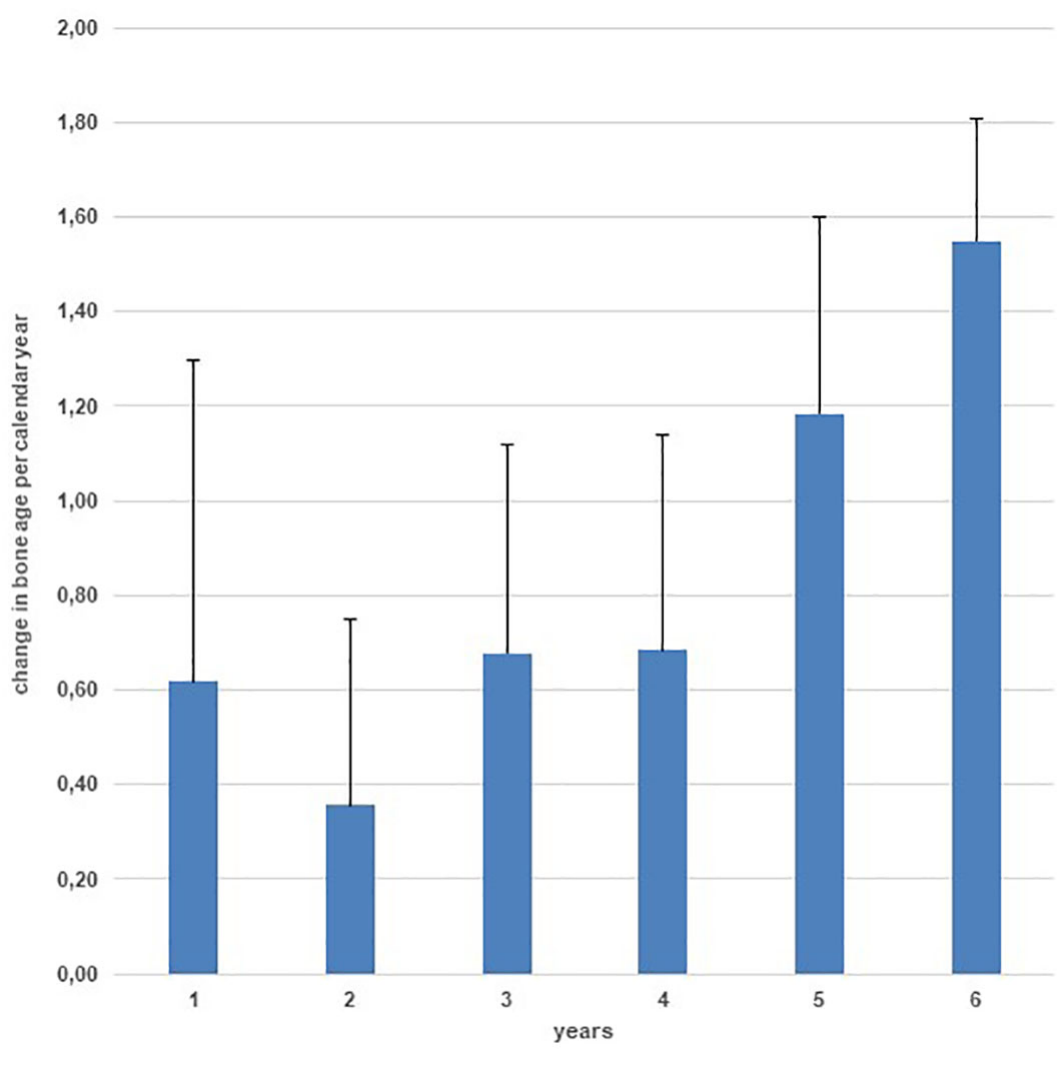
Yearly bone age progression during treatment and during 2 years of follow-up.

Mean ( ± SD) bone age was 10.4 ± 0.6 years at start of treatment and 12.8 ± 0.6 years at end of treatment. During the 4-year treatment, bone age increased only 2.4 ± 0.5 years resulting in a mean annual increment of 0.6 years/calendar year, while during the follow-up period, bone age increment accelerated to 1.3 years/calendar year (**Figure 4**).

### 3.5 Puberty evolution

Puberty resumed immediately after withdrawal of the GnRHa therapy and menarche occurred in all participants within 2 years after GnRHa stop.

### 3.6 Safety parameters, adverse events, and serious adverse events

Mean serum IGF-1 levels peaked to maximum levels after 2 years of treatment (+2 SD according to center-specific reference ranges) and decreased during further treatment to reach a mean of 0 SD one-year post treatment.

Fasting insulin levels increased 2.5-fold during treatment from 6.42 mU/l ± 2.87 at start, to a maximum of 15.16 mU/l ± 11.96 after 1 year. Nonetheless fasting glucose and HbA1c levels remained within the normal range. After stop of treatment, fasting insulin levels normalized. One patient had overweight at start of treatment and insulin resistance, but normal fasting glucose levels and normal Hb1Ac levels, which remained stable throughout the study.

Clinical adverse events consisted of injection site reactions (pain, bruising, scarring) and common health problems for this age group (**Table 3**).

**Table 3:** Adverse events according to body system occurring more than twice.

Body system	Diagnosis	Total
**respiratory, thoracic and mediastinal disorders**	bronchitis	3
	asthma	6
	tonsillitis/pharyngitis	11
	upper airway infection	26
	otitis media	3
**gastro-intestinal disorders**	nausea	8
	gastro-enteritis	14
**infections and infestations**	fever	4
**nervous system disorders**	headache	13
**musculoskeletal and connective tissues disorders**	fractures	2*
	tendinitis	4
**endocrine disorders**	insulin resistance	1*

*Fractures and insulin resistance were added because they are possibly treatment related.

Three serious adverse events (SAE) were reported: one patient had an exacerbation of a pre-existing asthma, requiring hospitalization. Another participant developed a fracture of her metatarsals during an intensive ballet training (this SAE was considered as possibly related to the intervention), and a third patient was hospitalized for a major depression during the follow-up phase, 2 years after the stop of treatment.

## 4 Discussion

In this multicenter, open-label, case-control study we evaluated a 4-year GH/GnRHa combination therapy in early pubertal girls with a PAH below -2.5 SDS and compared the results to untreated historical controls.

Mean AH in girls after 4-year treatment with GH/GnRHa was 6.4 cm higher than in matched controls despite the fact that PAH was higher in the control group. We also found that AH surpassed initial PAH by 12.0 ± 2.6 cm in treated girls and by 4.2 ± 3.6 cm in controls. Only a few studies reported the effect of GH/GnRHa combination treatment on AH in girls with ISS. Van Gool et al. reported a gain in AH *vs* PAH of 7.4 cm (20) and Maniati Christidi et al. of 5.7 cm (21). In a randomized controlled trial with GH versus GH/GnRHa, Pasquino et al. reported a gain of AH *vs* PAH of 6.1 cm with GH alone and of 10.0 cm with the GH/GnRHa combination treatment (19). In a retrospective study, Lazar et al. saw a gain of AH *vs* PAH of 7.2 cm with GH and 9.5 cm with GH/GnRHa in pubertal ISS girls (23). Benabbad et al. found no difference between GH alone and GH/GnRHa combination treatment in ISS patients when initiated at puberty onset (27). In our study we did not have a GH-only arm, so we are unable to measure the contribution of each treatment component separately. A meta-analysis and systematic review on the effect of GH-only treatment in ISS revealed a treatment effect of 4-6 cm versus untreated controls and about 4 cm compared with PAH (39, 40). The large treatment effect in our study supports the notion that concomitant GnRHa treatment adds to the effect of GH on height gain.

An important finding in the control group is that AH surpassed PAH by 4.2 ± 3.6 cm. The comparison between AH and PAH, heavily relies on the accuracy of the AH prediction. To establish the prediction error, we compared AH to the PAH in a matched control group and found that the Greulich and Pyle-Bayley and Pineau method underestimated AH. In most studies, AH prediction in untreated ISS patients was reported to be very accurate albeit with a wide prediction error (41–45). However, other authors found an average under-prediction of 2.8 cm and 2.3 cm in cohorts of girls comparable to ours (46, 47). This may be explained by the observation that the accuracy of AH prediction in ISS patients depends on the degree of bone age delay at the time of the prediction. If the bone age delay is close to 2 years, the AH prediction matches the measured AH, but if there is minimal bone age delay, as in the present study, under-prediction is expected (46).

Another important finding is the fact that PAH at the end of treatment overestimated AH. PAH continued to rise during the treatment phase because the bone age progression was kept at a low pace during the entire treatment period. When the GnRHa treatment stopped, bone age accelerated significantly. As a result, PAH at the end of the treatment overestimated AH by a mean of 2.6 cm. This was also demonstrated in the Dutch ISS study, which showed a gain in PAH of 7.8 cm between treated and untreated girls after the 3-year treatment period (25), however at AH the gain was only 5.5 cm (20).

In our study the PAH after 4 years of treatment was the same as the PAH after 3 years of treatment, suggesting that a fourth year of GH/GnRHa treatment might not have a major contribution to AH. On the other hand, the fourth treatment year added more cm in actual height and reduced the post treatment period that normally results in a loss of height SDS due to a fast bone maturation. The comparison of results between a shorter and longer treatment duration published in the literature is in favor of a longer treatment period (19) but only a comparative trial can resolve this question.

Most treated girls reached a normal height > -2 SDS (87.5%) and reached a height equal or more than TH (68.7%), while the minority of the controls did (37.5% and 6.2%, respectively). Despite these promising results, one must ask whether the number of girls attaining a normal height justifies the cost of the treatment protocol. Further research with larger study populations might be required to establish the cost-effectiveness of this treatment.

In our study we did not find an effect of birth weight and length, gestational age, maternal height, paternal height, TH, height deficit in relation to TH, nor bone age at onset on the outcome parameters AH, AH-PAH, and AH-H_0_. However, the negative correlation between H_0_ SDS and height gain SDS, and the positive correlation between bone age delay at start and height gain SDS, suggests that girls with a smaller height at start and a more delayed bone age could benefit more from this treatment. With a small sample size, however, caution must be applied, and these findings must be confirmed in larger studies.

The safety profile of the GH/GnRHa combination treatment in this study was acceptable. Most adverse events were typical for this age group of active adolescents. One adverse event possibly related to the intervention was reported: a fracture of metatarsals in a ballet dancer. Benabbad et al. also reported that bone fractures occurred more frequently in the combined GH/GnRHa group than the GH-alone group, and that they were associated with an abnormally low BMD (27). It has been reported that GnRHa treatment (with or without GH) decreases bone mineral density (BMD) in children with short stature (7, 48, 49), but that BMD returns to normal after cessation of therapy (20, 50). Future research is needed to better understand the risk of decreased BMD associated with this GH/GnRHa treatment. Another concern is the reduction in insulin sensitivity due to GH therapy. Therefore, fasting insulin and glucose levels and HbA1c levels were monitored during the treatment period. The observed increase in fasting insulin levels was not associated with abnormal fasting glucose nor HbA1c levels; fasting insulin levels normalized after cessation of treatment. This agrees with the findings of the Dutch study group that GH/GnRHa combination therapy did not adversely affect glucose metabolism in SGA children (51, 52).

The key strengths of this trial are its prospective and rigorous experimental design, with a uniform treatment schedule, a long GH/GnRHa treatment duration, a homogeneous study population and a follow-up period until AH. In contrast to most previous studies, treatment with GnRHa and GH was started simultaneously. The patients in our study were treated with a GH dose of 50µg/kg/day, since Wit et al. showed a dose effect for GH in ISS patients (46) and in a dose response GH/GnRHa trial in SGA children, Lem et al. demonstrated a significantly better outcome with the higher GH dose in combination with GnRHa (24). In some studies, GH treatment was continued until near AH. This could have further increased AH, but the effect would probably be limited by the acceleration of bone age and fast closure of the growth plates after stopping GnRHa. To maximize the effect of GH and to obtain a uniform treatment schedule, we choose a 4-year combination treatment duration. After 4 years, most of the pubertal height gain was obtained and we did not see a decrease in height SDS after stopping treatment, as seen in studies with a shorter treatment duration. In addition, the PAH after 3 and 4 years of treatment was similar, suggesting that a three-year treatment period might result in equal height gain, and reduce the number of dropouts. This hypothesis should be explored in a future randomized comparative trial. Moreover, with such a long treatment duration, treatment adherence sometimes became a problem due to the large number of injections for a long period of time, and the psychological effect of postponing puberty for 4 years. This was apparently less tolerated by boys than by girls, as was demonstrated by the failure to include enough boys and the high drop-out rate.

To obtain a homogeneous study population, only girls with an adult height prediction of less than -2.5 SDS for the Flemish population were included. This study group predominantly included girls with ISS, although familial short stature and short stature following SGA was not excluded. Most girls were early pubertal, and this may contribute to the positive outcome of this study. Some studies included girls with a more advanced pubertal development. A more advanced puberty stage, or a small size at birth may reduce the treatment effect and therefore underestimate the efficacy of this combination treatment in puberty stage B2 girls without SGA. Our *post hoc* analysis removing and replacing these patients did not substantially change the results. However since there were only 2 patients with a B3 stage and only 3 SGA patients in the control group, this study was not powered enough to answer this question.

Furthermore, one of the major strengths of this study is that we followed the treated girls until AH. Some studies report only on PAH at end of treatment. This may lead to overestimation of treatment results as again demonstrated in our study. Our results based on measured AH better reflect the true treatment effect.

This study has certain limitations. A first limitation is the nonrandomized design, the inclusion of a selection of historical controls and the relatively small study population. One third of the participants dropped out due to low compliance. Importantly, as demonstrated in **Table 1**, our analysis shows that the excluded patients did not respond differently from the PP group in terms of response, excluding a selection bias. Our small study should encourage the conduction of a large, randomized, placebo controlled study to firmly establish the value of this treatment in early pubertal girls with a poor adult height prediction.

An additional limitation of our study is the lack of a centralized bone age reading, which may have led to inter–observer variances. This problem could be avoided by using an automated bone age estimation, which is unfortunately not available in all hospitals. A last limitation is the lack of quality-of-life assessment. Due to the long treatment protocol some girls were relatively old before puberty started and a future study should evaluate the effects on quality of life of postponing puberty for 4 years, to weigh the effectiveness of treatment against the psychological burden that it may entail.

Taken together, our findings suggest that a 4-year combination treatment of GH and GnRHa results in a clinically relevant and statistically significant increase in AH in early pubertal GH-naïve girls with a poor adult height prognosis. The impact of increasing AH must be weighed against the financial and the psychological burden of this intensive treatment. Moreover, the long-term effect on bone health remains to be further explored.

## Data availability statement

The raw data supporting the conclusions of this article will be made available by the authors, without undue reservation.

## Ethics statement

The studies involving human participants were reviewed and approved by Etisch Comité UZA University Hospital Antwerp Edegem Belgium. Written informed consent to participate in this study was provided by the participants’ legal guardian/next of kin.

## Author contributions

HD: First authorship. AF, CH, ST, CB, M-CL, KD, GM, ML, IG, JV, CD, MZ, JD, VB: co-authorship. SV: senior authorship. RR and MD: Equal contribution and last authorship. All authors contributed to the article and approved the submitted version.
