# Supplementary material for: Efficacy and safety of a 4-year combination therapy of growth hormone and gonadotropin-releasing hormone analogue in pubertal girls with short predicted adult height
Source: Front Endocrinol (Lausanne). 2023 Mar 17;14:1113750. doi: 10.3389/fendo.2023.1113750 (PMC10064858; doi:10.3389/fendo.2023.1113750)
Supplement: Supplementary file 1 [file Table_1.docx]

*Supplementary table 1. Literature overview of combined GH and GnRHa studies. CA: chronological age; BA: bone age; PAH: predicted adult height; AH: adult height; NAH: near adult height; ISS: idiopathic short stature; CPP: central precocious puberty; GHD: growth hormone deficiency; EP: early puberty; SGA: small for gestational age.*

| Author | Design | Treatment | Duration | Subjects | Before treatment  CA BA Height H-SDS PAH | | | | | After treatment  AH  (AH-SDS) |
| --- | --- | --- | --- | --- | --- | --- | --- | --- | --- | --- |
| Balduci 1995  (16) | GH+GnRHa | GH 0.033 mg/kg/d 6d/w  + GnRHa 3.75 mg/28d | 2Y | 10 ♀ ISS | 11.6±1.4 | 10.6±0.9 | 127.4±5.5 | -2.7±0.7 | 143.2±3 | 144.6±3  (-2.8±0.8) |
| Lanes 1998  (17) | GH+GnRHa  Controls | GH 0.033 mg/kg/d 6d/w  + GnRHa 3.75 mg/28d  No treatment | 2Y | 10 ♂+♀  ISS | 11.8±1.3  11.4±1.0 | 11.2±0.9  11.0±0.8 | 128.9±7.5  128.9±7.8 | −2.4±0.4  −2.3±0.4 | 150.7±9.8  151.8±10.1 | 151.7±2.4  (−2.7±0.6)  150.3± 9.8  (−2.9±0.8) |
| Pasquino 1999 (10) | GH+GnRHa  GnRHa | GH 0.033 mg/kg/d  + GnRHa 3.75 mg/28d  GnRHa 3.75 mg/28d | 3Y | 20 ♀ CPP | 10.0±0.5  7.6±0.2 | 12.0±0.2  10.4±0.3 | NA  NA | −1.5±0.2  −1.0±0.3 | 152.7±1.7  155.5±2.0 | 160.6±1.3  (0.2±0.2)  157.1±2.5  (−0.4±0.3) |
| Mericq 2000 (15) | GH+GnRHa  GH | GH 0.033 mg/kg/d  + GnRHa 3.75 mg/28d  GH 0.033 mg/kg/d | 3Y | 21 ♂+♀  GHD | 14.2±0.5  14.0±0.4 | 11.5±0.4  11.0±0.3 | NA  NA | -4.0±0.4  -4.0±0.3 | NA  NA | NA  (NAH -1.3±0.5)  NA  (NAH -2.7±0.3) |
| Pasquino 2000 (19) | GH+GnRHa  GH | GH 0.05 mg/kg/d 6d/w  + GnRHa 100mcg/kg/21d  GH 0.005 mg/kg/d 6d/w | 4.5Y | 24 ♂ ISS | 10.2±0.9  10.7±1.0 | 10.6±1.9  10.1±1.4 | NA  NA | NA  NA | 146.3±5.0  145.6±4.4 | 156.3±5.9  151.7±2.7 |
| Kamp 2001  (25) | GH+GnRHa  Controls | GH 0.050 mg/kg/d + GnRHa 3.75 mg/28d  No treatment | 3Y | 16 ♀ ISS | 11.5±0.1  11.5±0.1 | 10.9±0.2  10.9±0.2 | NA  NA | -2.0±0.1  -2.0±0.1 | 151.8±1.0  151.8±1.0 | NA  (PAH after 3Y +10.4 cm)  NA  (PAH after 3Y +2.4 cm) |
| Maniati-Christidi 2003 (21) | GH+GnRHa | GH 0.020 mg/kg/d + GnRHa 3.75 mg/28d | 3Y | 8 ♀ ISS | 10.5±0.8 | 10.1±1 | 129.3±5 | NA | 148.8±2.6 | 154.5±3.6 |
| Tauber 2003  (14) | GH+GnRHa  GH | GH 0.020-0.023 mg/kg/d 6d/w  +GnRHa 1.5-3 mg/28d  GH 0.020-0.023 mg/kg/d | 1-3 Y | 14 ♀  GHD | 11.5±1.5  11.5±1.6 | 9.5±1.7  9.7±1.5 | 128.5±7.7  126.5±6.4 | -2.6±0.5  -2.8±0.8 | 147.8±5.0  148.8±5.3 | 152.5±3.1  (-1.8±0.5)  152.0±6.0  (-1.8±1.0) |
| Tuvemo 2004 (30) | GH+GnRHa  GnRHa | GH 0.033 mg/kg/d  + GnRHa 6x300 mcg IN/d  GnRHa 6x300 mcg IN/d | 2-4Y | 46 ♀ Adopted, EP  CPP | 8.4±0.78  8.2±0.83 | NA  NA | 132.0±5.5  130±7.3 | 0.5±1.1  0.3±0.9 | NA  NA | 158.9±5.3  (-1.1±0.8)  155.8±6.8  (-1.6±1.0) |
| Mul 2005  (29) | GH+GnRHa  GH | GH 1.33 mg/m /d  + GnRHa 3.75 mg/28d  GH 1.33 mg/m /d | 3Y | 26 ♂+♀ Adopted, EP | 9.6±0.9  9.6±0.9 | 11.6±0.8  10.7±1.1 | 150.0±4.9  145.7±7.7 | -0.66±0.9  -0.89±1.0 | 146.8±4.8  149.8±5.6 | 155.0±5.5  (-2.1±0.9)  155.0±5.6  (-2.1±0.9) |
| Van Gool 2007 (20) | GH+GnRHa  Controls | GH 0.050 mg/kg/d + GnRHa 3.75 mg/28d  No treatment | 3Y | 32 ♂+♀  ISS, SGA | 11.8±0.7  11.6±0.7 | NA  NA | 135.1±4.5  136.1±4.5 | -2.4±0.5  -2.5±0.5 | 157.4±9.8  160±10.1 | 161.8±6.3  (-2.0±1.0)  159.5±5.5  (-2.3±0.6) |
| Scalco 2010  (31) | GH+GnRHa  Controls | GH 0.050 mg/kg/d + GnRHa 3.75 mg/28d  No treatment | 2-4.9Y | 10 ♂+♀ SHOX | 11.8±2.1  11.4±1.4 | NA  NA | NA  NA | -2.3±1.3  -1.2±0.7 | NA  NA | NA  (-2.4±0.6)  NA  (-1.2±0.7) |
| Colmenares 2012 (53) | GH+GnRHa  GH | GH 0.033-0.050 mg/kg/d  + GnRHa 3.75 mg/28d  GH 0.033-0.050 mg/kg/d | 2Y | 32 ♂+♀  ISS | 12.0±1.6  12.2±1.6 | 11.4±0.9  10.9±1.9 | NA  NA | -2.3±1.1  -2.3±0.9 | 153.7±10  160.8±10.2 | NA  (NAH -1.6±1.5)  NA  (NAH -0.1±1.9) |
| Jung 2014  (13) | GH+GnRHa  GnRHa | GH 0.033 mg/kg/d , 6d/w  +GnRHa 75–150 μg/kg/28d  GnRHa 75–150 μg/kg/28d | 0.5-4Y | 82 ♀ CPP | 8.8±5.5  8.7±0.7 | 10.5±0.8  10.5±0.8 | 133.8±5.4  136.9±7.4 | 0.7±0.8  1.5±1.0 | 154.6±2.5  156.6±3.9 | 159.3±5.3  (0.0±1.0)  160.4±4.2  (0.4±0.8) |
| Gyon 2015 (12) | GH+GnRHa  GnRHa | GH 0.7 ± 0.1 IU/kg/wk  + GnRHa 3.75 mg/28d  GnRHa 3.75 mg/28d | 1-2Y | 85 ♀ CPP | 7.9±0.7  8.2±0.8 | 10.4±1.3  10.5±1.1 | NA  NA | 0.3±1.3  0.9±1.0 | 147.7±8.0  153.7±7.4 | (0.4±1.0)  (0.8±1.0) |
| Benabbad 2018 (27) | GH+GnRHa  GH | GH 0.050 mg/kg/d  + GnRHa 11.25mg/3M  GH 0.050 mg/kg/d | 2.4Y | 35 ♂+♀  ISS | 12.1±1.4  12.1±1.3 | 10.8±1.6  11.0±1.5 | NA  NA | -2.5±0.5  -2.5±0.5 | -2.7±0.8 (SDS)  -2.6±0.9 (SDS) | NA  (NAH -2.7 SDS)  NA  (NAH -2.6 SDS) |
| Lazar 2019 (23) | GH+GnRHa  GH | GH 0.050 mg/kg/d  + GnRHa 3.75 mg/28d  GH 0.050 mg/kg/d | 2-4Y | 30 ♀ ISS | 11.3±1.2  12.7±0.8 | -0.6±1.2  (SDS)  -1.6±0.5 (SDS) | NA  NA | -2.1±0.7  -2.6±0.7 | 146.0±3.1  148.1±2.8 | 155.5± 3.3  (-1.06±0.5)  155.3±3.6  (-1.9±0.5) |
| Li 2020 (26) | GH+GnRHa | GH 0.045 mg/kg/d  + GnRHa 3.75 mg/28d | 2Y | 12 ♀ ISS | 12.6±1.1 | 12.8±1.8 | NA | -3.6±1.1 | 146.0±6.5 | 156.8±6.4  (-0.33±1.9) |
