# Supplementary material for: Efficacy and safety of a 4-year combination therapy of growth hormone and gonadotropin-releasing hormone analogue in pubertal girls with short predicted adult height
Source: Front Endocrinol (Lausanne). 2023 Mar 17;14:1113750. doi: 10.3389/fendo.2023.1113750 (PMC10064858; doi:10.3389/fendo.2023.1113750)
Supplement: Supplementary file 2 [file Table_2.docx]

*Supplementary table 2: Sensitivity analysis: clinical characteristics and outcome parameters in treated girls in comparison to matched controls without SGA and puberty stage B3 patients. (mean ±SD). TH: target height; Ho: height at the start of treatment; BA: Bone age; AH: adult height; PAH: predicted adult height*

| **Clinical characteristics** | **GnRHa +GH treated girls (n=15)** | **Matched controls (n=15)** | **P** |
| --- | --- | --- | --- |
| **Age at start (y)** | 11.0 ± 1.3 | 11.6 ± 0.9 | 0.173 |
| **Birth Length (cm)** | 46.9 ± 1.7 | 46.9 ± 2.7 | 0.967 |
| **Birth Weight (g)** | 2794 ± 302 | 2724 ± 643 | 0.619 |
| **Gestational age (w)** | 38.5 ± 1.6 | 39.0 ± 2.6 | 0.519 |
| **TH (cm)**  **(SDS)** | 157.4 ± 5.4  -1.4 ± 0.8 | 159.3 ± 4.6  -1.2 ± 0.7 | 0.331  0.282 |
| **H0 (cm)**  **(SDS)** | 131.4 ± 4.2  -2.2 ± 0.7 | 132.5 ± 4.4  -2.5± 0.6 | 0.467  0.198 |
| **H0- TH (cm)**  **(SDS)** | -26.0 ± 4.0  -0.6 ± 1.1 | -27.0 ± 6.4  -1.3 ± 1.0 | 0.637  0.139 |
| **BA at start (y)** | 10.6 ± 0.6 | 10.6 ± 0.6 | 0.967 |
| **PAH (cm)**  **(SDS)** | 147.8 ± 2.0  -3.1 ± 0.3 | 149.4 ± 2.4  -2.8 ± 0.4 | 0.058  0.059 |
| **AH (cm)**  **(SDS)** | 159.9 ± 4.6  -1.1 ± 0.8 | 153.5 ± 3.3  -2.1 ± 0.6 | <0.001  <0.001 |
| **AH-PAH (cm)**  **(SDS)** | 12.1 ± 4.0  2.0 ± 0.7 | 4.1 ± 3.2  0.7 ± 0.5 | <0.001  <0.001 |
| **AH-TH (cm)**  **(SDS)** | 2.1± 5.4  0.4 ± 0.8 | -6.2 ± 4.1  -0.8 ± 0.8 | <0.001  <0.001 |
| **AH– H0 (cm)**  **(SDS)** | 28.5 ± 4.4  1.0 ± 1.0 | 20.9 ± 4.7  0.4 ± 0.7 | <0.001  0.037 |
